# Tuina Massage Improves Cognitive Functions of Hypoxic-Ischemic Neonatal Rats by Regulating Genome-Wide DNA Hydroxymethylation Levels

**DOI:** 10.1155/2019/1282085

**Published:** 2019-10-23

**Authors:** Yunpeng Zhang, Chao Gao, Danmei Chen, Cuiting Wang, Long Chen, Yaodong Zhang, Bing Li

**Affiliations:** ^1^Research Center for Clinical Medicine, Jinshan Hospital, Fudan University, Shanghai, China; ^2^Institute of Neurology, Academy of Integrative Medicine, Fudan University, Shanghai, China; ^3^Department of Rehabilitation, Henan Children's Hospital, Zhengzhou University, Zhengzhou, China; ^4^Department of Neurology, Jinshan Hospital, Fudan University, Shanghai, China; ^5^Department of Pediatrics, Henan Children's Hospital, Zhengzhou University, Zhengzhou, China

## Abstract

In addition to abnormalities of motor and posture, children with cerebral palsy (CP) often have intellectual disability. As a complementary and alternative traditional Chinese medicine (TCM) therapy, Chinese Tuina massage, also called Tuina in China, has been widely applied in clinical treatment for CP in China for a long time. However, the molecular basis for this still remains largely unknown. Recently, DNA hydroxymethylation has been shown to be sensitive to environment and plays critical roles in some neurological disorders, whereas the research focusing on the relationship between 5 hmC and Tuina therapy for cerebral palsy is deficient. In our study, we first observed that Tuina improved learning and memory functions of hypoxic-ischemic (HI) rat pups. Meanwhile, 5 hmC level of the temporal lobe cortex in the HI neonatal rat model is decreased significantly compared to that of the rats in control and Tuina groups. Then, we used the hMeDIP-Seq method to explore whether and how DNA hydroxymethylation is involved in Tuina therapy for cerebral palsy. Genomic annotation of DhMRs of HI group's hypo-hydroxymethylation to genes revealed enrichment in multiple neurodevelopmental signaling pathways. Moreover, we found the depletion of 5 hmC modifications in genes associated with neuronal development was accompanied by reduced mRNA levels of these genes. Taken together, our results indicate that Tuina may regulate the expression of neurodevelopment-related genes by changing the status of DNA hydroxymethylation, thereby improving learning and memory functions of cerebral palsy.

## 1. Introduction

Cerebral injury occurring during the perinatal period is a major cause for acquired disability. The occurrence of cerebral injury in infants is correlated with multiple factors, such as hypoxia-ischemia, infection, oxidative stress, and inflammatory response [1, 2] among which hypoxia-ischemia (HI) may induce cerebral palsy (CP) by triggering perinatal cerebral lesions in preterm [3]. CP is the commonest neonatal chronic disability, characterized by motor and postural impairments, and is often accompanied by cognitive and learning deficits [4]. In basic science studies, the HI neonatal rat model has been widely used in exploring the behavioral outcomes and pathogenesis of cerebral palsy [5–7]. Despite the great advancement in CP studies, the molecular mechanisms of how hypoxia-ischemia contributes to CP development and progression still stay unclear.

To this day, the current therapies for CP mainly includes orthopedic surgery, antispasticity medications, and various kinds of motor learning interventions [8], most of which merely work to treat the complications related to CP and may bring about side effects. However, traditional Chinese medicine (TCM), including Tuina, acupuncture, and herbal medicine, alone or combined with conventional therapy of western medicine, has been widely used as an alternative treatment for CP in China [9]. Tuina is an ancient form of medical massage in Chinese medicine, with applied finger pressure to acupoints that are putatively sensitized by organ impairment [10]. Notably, Tuina, as a safer and more effective intervention, has displayed obvious effects on cerebral palsy in clinical practice. However, the exact mechanism of how Tuina works on CP has not yet been elucidated.

On the contrary, pathogenically, environment-sensitive epigenetic modifications have already been proven to be involved in neurogenesis [11], learning and memory [12], and synaptic plasticity [13]. Among various epigenetic modifications, DNA methylation on the fifth carbon of cytosine (5-methylcytosine, 5 mC) is the most extensively studied epigenetic modification which plays important roles in chromatin structure remolding, transcriptional suppression, and cellular differentiation [14–16]. Furthermore, oxidation of 5 mC into 5-hydroxymethylcytosine (5 hmC) by the ten-eleven translocation (TET) gene family is proposed as a novel mechanism for removal of 5 mC [17, 18], and 5 hmC has been suggested to be critical in maintenance of neuron structure and functions due to its obvious enrichment in the brain [19–21]. Meanwhile, 5 hmC alteration is also identified by many studies to be associated with neurological disorders, such as Alzheimer's disease [22], Rett syndrome [23], fragile X-associated tremor/ataxia syndrome [24], Huntington's disease [25], and the autism spectrum disorders [26]. These evidences provided the basis for the hypothesis that 5 hmC alterations may play critical roles in nervous system disease.

To explore the presence of the involvement of Tuina therapy in the alteration of 5 hmC in CP, we applied an established chemical labeling and affinity purification method coupled with high-throughput sequencing technology to reveal the possible genome-wide 5 hmC alterations in HI rats prior to and after Tuina treatment in order to seek the relationship between 5 hmC and Tuina therapeutic effects on CP, thereby providing evidence to support application of Tuina therapy for cerebral palsy.

## 2. Materials and Methods

### 2.1. Animal Model

All experiments were approved by the Ethics Committee for Animal Care of Jinshan Hospital affiliated to Fudan University. A modified Rice–Vannucci model was used as previously described [27, 28]. The neonatal HI model was performed on Sprague-Dawley male rat pups (Slac Laboratory Animal Company, Shanghai, China) on postnatal day 3 (P3) via ligation of the left common carotid artery under 3% isoflurane anesthesia. Surgery time for each rat was controlled within 10 minutes. After recovery for 1 hour, these pups were placed in a hypoxia chamber at 37°C and subject to the gas mixer composed of 8% O_2_ and 98% N_2_ for 3.5 hours. Sham animals had the same operation to expose the common carotid artery without ligation and hypoxia. After surgery, the rat pups were returned to their home cage.

### 2.2. Tuina Treatment

Tuina treatment was performed by a specialized researcher with professional physician certificate. From the day of P4 to P31, rat pups in the Tuina group accepted Tuina intervention which is carried out 120 times/min for 15 minutes, once a day. The procedure of Tuina is as follows: first, the rat was placed on the palm of the manipulator's left hand to adjust to the environment; secondly, the manipulator used the middle finger of his right hand to gently rub the rat from the neck to tail three times to relax its muscles; thirdly, Tuina was carried out by successive rubbing, kneading, and one-finger pushing along the spine of the rats from top to bottom and from inside out on dorsum and ambilateral ligaments as well as the muscles along the du meridian and bladder meridian of the rat with reference to related traditional Chinese medicine (TCM) works.

### 2.3. Righting Reflex

Righting reflex of neonatal rats was assessed from P5 to P11, as described before [29, 30]. During this test, the rat pups were placed in a supine position on a cleaned flat surface and the time for these pups to reachieve the prone position with all four paws was measured.

### 2.4. Gait Reflex

Rat pups were placed in the center of a white rounded cardboard (13 cm in diameter), and the day when they moved off the circle with both forelimbs was recorded [31].

### 2.5. Inclined Plane Test

**Figure 1:**
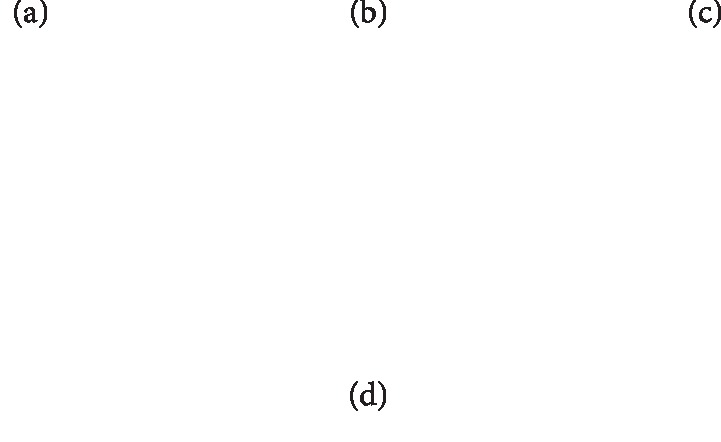
Tuina improves hypoxia-ischemia-induced weight loss, limb strength weakness, and neurological reflexes disorder: (a) average weights of rat pups (*n* = 11) starting from the 4th day after birth (P4) (i.e., 1 day after the hypoxic-ischemic insult); (b) angle of the inclined plane test on P21 (*n* = 11); (c) the postnatal day of gait reflex (*n* = 11); (d) righting reflex time (*n* = 11) (^*∗*^vs. control group, *p* < 0.05; ^#^vs. HI group, *p* < 0.05).

This test was used to measure limb strength by recording the maximal angle of the slope onto which the rat pups can still manage to grip on the day of P21, as shown in Figure 1(b) [32]. During this test, the rats were placed facing right or left and the angle of the plane was increased by 5° every five seconds in order to determine the maximal angle of the slope to which the rat could still hold [33].

### 2.6. Step-Down Avoidance Test

This test was implemented to measure the retention of memory [34, 35]. A rat was gently placed on a cylindrical insulation platform (4.5 cm in diameter and height) in a conditioning chamber to adapt to the surrounding environment for 3 min. Once the rat steps down the platform, it will be electrified immediately. In the 5-minute-training session (P26), the rats were trained to jump back onto the platform to avoid the electric stimulation. In the test sessions (24 h after training, i.e., P27), the latency time (the time for the rats to step down from the platform for the first time) and the number of errors (frequency of the rats stepping down from the platform) were recorded.

### 2.7. Morris Water Maze (MWM) Test

In order to evaluate spatial learning and memory, the Morris water maze test was performed for 5 days as previously described [36, 37]. The water maze (Shanghai Xinyuan Information Technology Company, Shanghai, China) was divided into four quadrants, and a platform (5 cm in diameter) was placed 1 cm below the water surface in one quadrant. In the training sessions (P27–P30), all the rats were trained four times a day for four consecutive days, and they were allowed to find the hidden platform within 60 s. If the rat failed to find the platform within 60 s, it was gently guided to the platform and allowed to stay on it for 30 s before being sent back to the cage. On the day for the spatial probe trial (P31), the platform was removed and the rats were released into the water facing the pool wall from the same position as in the training sessions. Subsequently, the motion tracks and the number of platform location crossings were recorded.

### 2.8. Dot Blotting

After completion of the above tests, the rat pups were sacrificed on P31 and their brains were dissected for collecting the total DNA of the left temporal cortex by a QIAamp DNA Mini Kit (Qiagen, Germany). 2.5 *μ*L genomic DNA (100 ng/*μ*L) was spotted on nylon membranes followed by baking at 80°C for 30 min to cross-link DNA [22]. After blocking with 5% nonfat milk for 1 h at room temperature, the membrane was incubated with polyclonal 5 hmC antibody (1 : 1000 dilution, Active Motif, United States, Cat. No. 39769) at 4°C overnight and the horseradish peroxidase (HRP)-conjugated secondary antibody was used to probe the next day. The dot blot membrane was stained with 0.02% methylene blue (MB) to ensure equal loading.

### 2.9. Hydroxymethylated DNA Immunoprecipitation (hMeDIP) Analysis and High-Throughput Sequencing

Genomic DNA was extracted from the left temporal cortex of rat pups (control: *n* = 3; HI: *n* = 3; Tuina: *n* = 3) and sonicated to 200–350 base pairs (bp). And the DNA fragments were end-repaired; A-tailed and Illumina adapters were ligated. Then, adapter-ligated DNA was denatured and incubated with 5 hmC antibody (Active Motif, United States, Cat. No. 39769) at 4°C overnight. Antibody-DNA complexes were captured by protein A/G beads. The immunoprecipitated DNA was purified and underwent high-throughput sequencing performed on the Illumina HiSeq3000 system (Illumina, United States) [38].

### 2.10. Mapping of Sequencing Data and Bioinformatics

Processing of sequencing data was performed as described previously [39]. FASTQ sequence files after biological duplications were aligned to UCSC rat reference genome (RGSC6.0/rn6) [40]. Unique, nonduplicate reads were used for peak calling and annotation by hypergeometric optimization of motif enrichment (HOMER) [41]. 5-hmC enrichment peaks were determined using a model-based analysis of ChIP-Seq (MACS) software [42]. And differentially hydroxymethylated regions (DhMRs) were identified among all pairs of 5-hmC-enriched samples by directly comparing one sample with another in every direction [43]. Gene ontology (GO) analyses were performed using the DAVID [44].

### 2.11. Quantitative RT-PCR

Total RNA was extracted from the left temporal lobe cortex. Reverse transcription of cDNA was performed using the PrimeScript RT Master Mix Kit (Takara, Japan). Quantitative RT–PCR reactions were performed on 7500 real-time PCR system (Applied Biosystems, United States) using SYBR green (Takara, Japan). *β*-actin was used as an internal control, and relative expression of target gene was determined by the 2^−ΔΔCT^ method. The sequences of primers used in the present experiment were listed as follows: Prkg2 primer (forward 5′-CCTCTGGATGTTCACCGCAAGAC-3′, reverse 5′-AGGTCATAGGTCCGGCTTGTGG-3′), Casd1 primer (forward 5′-GAGAGCAGACGGATGAATGGAAGG-3′, reverse 5′-AACAGATAAGCAGCCACCAGAACG-3′), Slc44a1 primer (forward 5′-ACACAGCCACAGCCATCAATAGC-3′, reverse 5′-CAGCCACTCGCAGAGCATTCTC-3′), and *β*-actin primer (forward 5′-CATGTACGTTGCTATCCAGGC-3′, reverse 5′-CTCCTTAATGTCACGCACGAT-3′).

### 2.12. Western Blotting Assay

The isolated left temporal lobe cortex was added with ice-cold protein lysate and centrifuged at 12000 r/min for 20 min at 4°C. Protein samples were separated by electrophoresis using SDS-PAGE and transferred onto PVDF membranes (Millipore, United States). After blocking with 5% nonfat milk for 2 h at room temperature, the membranes were incubated with the primary antibodies at 4°C overnight and then incubated with HRP-conjugated secondary IgG antibodies. The primary antibodies used here included anti-Tet1 (1 : 1000, Abcam, UK, Cat. No. 191698), anti-Tet2 (1 : 1000, Millipore, United States, Cat. # ABE364), anti-Slc44a1 (1 : 1000, Abcam, UK, Cat. No. 110767), and anti-*β* tubulin (1 : 2000, Cell Signaling Technology, United States, Cat. #2128). The membranes were then developed using ECL detection, and the signals were detected with Tanon-5200 Chemiluminescent Imaging System (Tanon, Shanghai, China). The protein levels were analyzed by Image J software (NIH, Bethesda, USA) and normalized to the corresponding *β*-tubulin level.

### 2.13. Immunofluorescence Confocal Microscopy

Rat pups were sacrificed on P31, and their hearts were perfused with 0.9% saline followed by 4% paraformaldehyde (PFA). After perfusion, the brains were dissected and fixed in 4% PFA overnight followed by being embedded in paraffin and made into 5 *μ*m serial sections. After microwave antigen retrieval in citric acid buffer at 95°C, nonspecific sites were blocked with 5% bovine serum albumin (BSA) for 1 h at room temperature. Next, the brain sections were incubated with 5 hmC primary antibody (1 : 1000, Active Motif, United States, Cat. No. 39769) overnight at 4°C followed by incubation with the secondary antibody (1 : 200, Life Technology, United States) 1 h at 37°C. 4′,6-diamino-2-phenylindole (DAPI) was used for nuclear immunofluorescence staining, and the immunofluorescence pictures were visualized and captured on a confocal microscope (Leica sp5, Germany).

### 2.14. Statistical Analysis

All the data were expressed as the mean ± standard error of the mean (SEM) and analyzed by using SPSS. One-way analysis of variance (ANOVA) was applied to determine the differences among the 3 groups, followed by nonparametric *t*-test and Tukey honestly significant difference test. *P* value <0.05 was considered statistically significant.

## 3. Results

Tuina improved weight loss, weakness of limb strength, and disorders of neurological reflexes induced by HI.

Body weight of rat pups was measured from P4 to P28, and it was found that, from P22 to P28, the body weight of the rats in the Tuina group (*n* = 11) increased significantly compared to the HI group (*n* = 11; Figure 1(a)). The results of the inclined plane test showed that the maximum angles were higher in the Tuina group than those in the HI group (Figure 1(b)), which indicated improvement in the weakness of limb strength in the Tuina group. The results of nervous system reflexes were as the following: in the gait reflexes test, the rat pups in the HI group started to move away from the circle with both forelimbs significantly later than those in control and Tuina groups (Figure 1(c)). In the righting reflex test, the rats in the Tuina group took shorter time to right themselves than those in the HI group from P7 to P11 (Figure 1(d)).

Tuina promoted learning and memory functions of hypoxic-ischemic rat pups.

**Figure 2:**
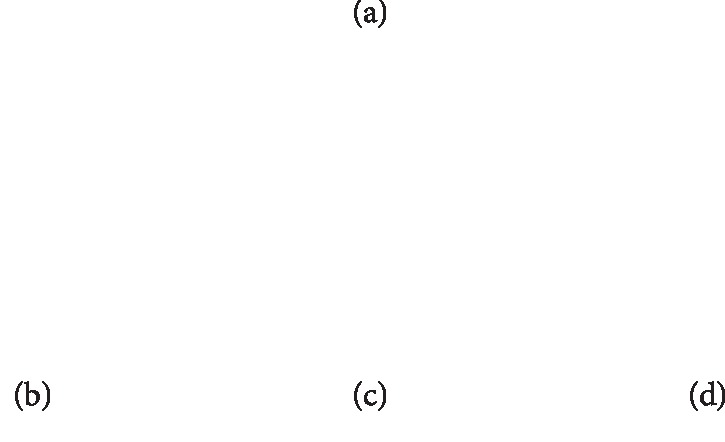
Tuina improves the learning and memory function in neonatal HI rats: (a) representative swimming paths of three groups (*n* = 11); (b) the average numbers of platform location crossings during the probe trial of the MWM test (*n* = 11); (c) the latency time of the step-down test (*n* = 11); (d) the errors number of the step-down test (*n* = 11) (^*∗*^vs. control group, *p* < 0.05; ^#^vs. HI group, *p* < 0.05).

Morris water maze test and step-down test were performed to evaluate the learning and memory functions of the rats. As shown in Figure 2(a), the motion tracks on the probe trial day of the MWM test show that the number of platform location crossings of the HI group was significantly less than that of the control group and Tuina group (Figure 2(b)). In the step-down test, the HI rats showed significantly shorter latency time and more errors compared to control and Tuina rats (Figures 2(c) and 2(d)).

Tuina increased 5 hmC and Tet2 levels in the cerebral temporal cortex of HI rats.

**Figure 3:**
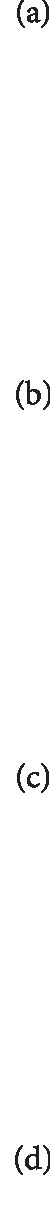
Reduced 5 hmC and Tet proteins level in the HI rat cortex. (a) Dot blotting analysis of genomic 5 hmC (*n* = 5). MB staining was used as a loading control. (b) Immunofluorescence staining in the left temporal cortex. 5 hmC was labeled with Alexa Fluor 594 (red), and the neuron nuclei were labeled with DAPI (blue) (Bars, 25 *μ*m). Western blotting results of Tet1 (c) (*n* = 5) and Tet2 (d) (*n* = 5) protein (^*∗*^vs. control group, *p* < 0.05; ^#^vs. HI group, *p* < 0.05).

To examine whether the level of 5 hmC was affected during CP development, we performed dot blot tests and observed that the 5 hmC level of HI rats decreased significantly compared to that of control, while this level was partially increased after Tuina (*P* < 0.05; Figure 3(a)). Meanwhile, immunofluorescence staining demonstrated the merging of 5 hmC and DAPI staining and HI led to decrease in genomic 5 hmC in the left temporal cortex, while Tuina treatment restored 5 hmC level to some degree (Figure 3(b)), which was in consistency with the results of dot blotting. Moreover, as shown in Figures 3(c) and 3(d), the HI group displayed decreased Tet1 and Tet2 expression compared to the control group, whereas only Tet2 was increased after Tuina therapy.

### 3.1. Genome-Wide 5 hmc Alteration and Distribution

**Figure 4:**
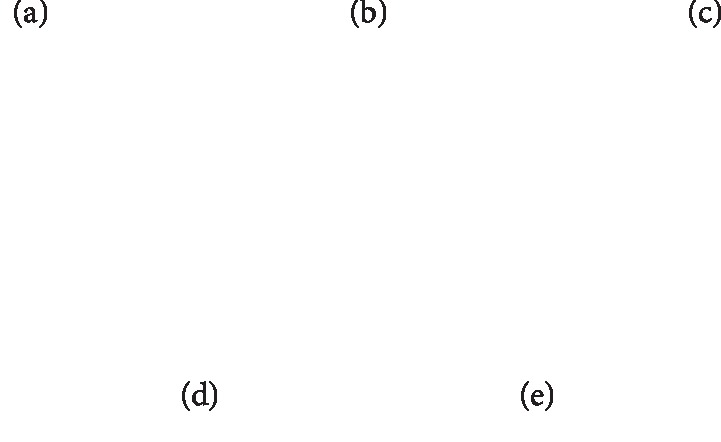
Genome-wide 5 hmc alteration and distribution. (a–c) Genome-wide 5 hmC reads density distribution. (d) Normalized 5 hmC densities overlapping with known genomic features. (e) Normalized 5 hmC read densities across TSS, TES, and RefSeq gene bodies. Gene bodies were normalized to 0–100% as relative positions.

To investigate the exact location and distribution of genome-wide 5 hmc, hydroxymethylated DNA immunoprecipitation (hMeDIP) followed by next-generation sequencing (hMeDIP-seq) [23] was carried out. The rats, respectively, from Control (*n* = 3), HI (*n* = 3), and Tuina (*n* = 3) groups were sacrificed on P31, and genomic DNA of the left temporal lobe cortex was extracted for hMeDIP-seq. Genome-wide pattern of 5 hmC level was evaluated by counting 5 hmC-mapped reads in every 10 kb bin from the samples of the control, Tuina, and HI groups and then normalized to the total sequencing coverage [24]. As shown in Figures 4(a)–4(c), the distribution of the genome-wide 5 hmC reads in the three groups was different, and then we further explored the specific gene regions in which these differences exist.  Figure 4(d) shows the overlapping features of normalized density of 5 hmC reads with the known genomic features on defined gene bodies and CGI (CpG islands) according to the UCSC table for RGSC6.0/rn6. Furthermore, as shown in Figure 4(e), the distributions of 5 hmC at gene bodies and 1 kb up- and downstream of gene bodies were also studied. It can be observed from Figures 4(d) and 4(e) that the 5 hmC level of the HI group differed from those of control group and Tuina group at the 5′ end of the genomic body, especially at the sites of ±500 bp of the transcriptional start sites (TSS) and the CGI within 500 bp of TSS.

### 3.2. Identification and Annotation of Differentially Hydroxymethylated Regions (DhMRs)

**Figure 5:**
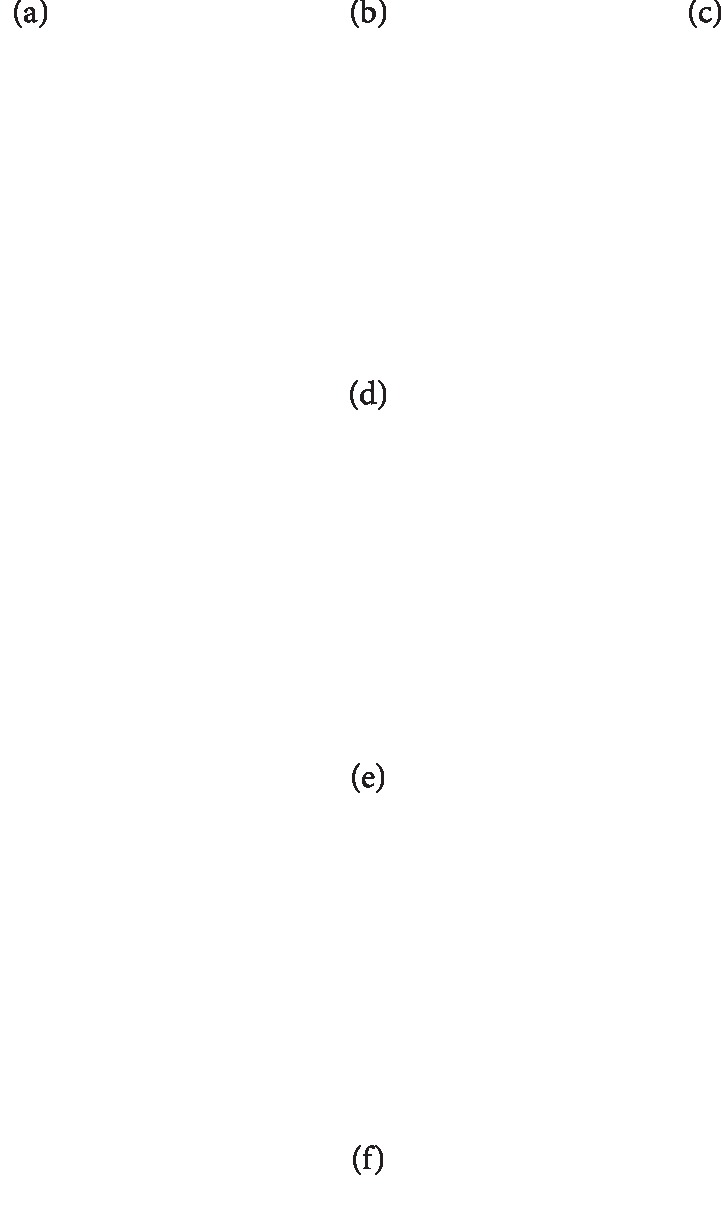
Identification and characterization of differentially hydroxymethylated regions (DhMRs). (a–c) Chromosome circular map of genome-wide DhMRs. Top ten GO enrichment analysis results of HI group's lower hydroxymethylation associated DhMRs compared to control (d) and Tuina (e) group. (f) RT-PCR analysis of the mRNA levels of selected hypo-DhMRs related genes. *β*-Actin was used as a control (^*∗*^vs. control group, *p* < 0.05; ^#^vs. HI group, *p* < 0.05).

To pinpoint the specific loci exhibiting altered 5 hmC profiles, we proceeded to identify and characterize the specific DhMRs of all autosomes and sex chromosomes across the genome. Interestingly, there was great difference in the distribution of hyper-DhMRs and hypo-DhMRs on the X chromosome (Figures 5(a)–5(c)). To further explore the biological significance of the found DhMRs, the genes associated with these DhMRs were extracted for gene ontology (GO) enrichment analysis. As shown in Figures 5(d) and 5(e), GO enrichment analysis indicated that these hypo-DhMRs in the HI group were found highly enriched on the functional pathways related to neurodevelopment and neuronal functions, including developmental cell growth, neuron projection extension, positive regulation of developmental growth, developmental growth involved in morphogenesis, and glial cell differentiation.

Based on the comprehensive analysis of hydroxymethylation differential genes lists of the three groups, we selected six hypo-DhMRs associated genes from the HI group which were all associated with the functions of nervous system to investigate the relationship between 5 hmC alterations and transcription levels of these genes. As what's expected, it was found out that, in accordance with the reduced 5 hmC modification in Casd1, Prkg2, and Slc44a1, the corresponding mRNA levels were also significantly decreased in the HI group in comparison with those in control and Tuina groups (Figure 5(f)).

## 4. Discussion

With general safety and reputed efficacy, traditional Chinese medicine (TCM) is readily accepted by the general population all over the world. TCM has been widely applied in various pediatric diseases, such as Ewing's sarcoma [45], asthma [46], Graves' disease [47], and pediatric allergic disorders [47]. Meanwhile, there are also studies indicating that TCM therapies including Tuina, herbal medicines, and acupuncture are well-tolerated and have positive therapeutic effects on cerebral palsy [9, 48–51]. Due to the promising clinical results and fewer side effects, Chinese Tuina massage has attracted growing interest in terms of the treatment for CP.

As a new epigenetic modification, 5 hmC not only serves as an intermediate of DNA demethylation processes but also acts as a stable epigenetic marker during development of various diseases [21]. 5 hmC is about tenfold more enriched in neurons than in other cells and its dynamic regulation is critical in postnatal neurodevelopment [20, 23, 43]. Moreover, the alteration of the global 5 hmC level has been considered closely related to many neurodegenerative disorders and neurodevelopmental diseases [22–26].

However, to the best of our knowledge, few studies have been focused on the connection between DNA hydroxymethylation and cerebral palsy, which became the inspiration for our research. In our study, the results of dot blotting and immunofluorescence revealed that the global 5 hmC level in the cerebral temporal lobe cortex was significantly decreased after HI injury, which is consistent with the reduced 5 hmC level in the mouse kidney after ischemia-reperfusion [52]. However, after Tuina treatment, 5 hmC level in the Tuina group was higher than that in the HI group. Furthermore, it is known that 5 hmC is produced via oxidation of 5 mC by Tet proteins, based on which we detected the levels of Tet1 and Tet2 proteins and found that both levels were significantly decreased after HI injury, whereas only Tet2 protein was increased after Tuina treatment. In all, these findings imply that the reduced Tet1 and Tet2 expression may contribute to the decrease in 5 hmC after HI injury and Tuina may increase 5 hmC level via enhancing Tet2 expression.

On the contrary, CGI methylation in promoters was found critical in gene silencing and 5 hmC may play a role in gene expression mediated by DNA demethylation [53, 54]. In our study, the HI group displayed decreased enrichment of genome-wide 5 hmC in CGI within 500 bp of TSS compared to control and Tuina groups, whereas Tuina increased genome-wide distribution of 5 hmC. Meanwhile, in line with the previous findings that the enrichment of 5 hmC in the gene body may has a strong correlation with the increased transcription of the concerned genes in neurons [19, 55], our RT-PCR results showed that the depletion of 5 hmC modification in Prkg2, Casd1, and Slc44a1 gene was accompanied by the reduced mRNA levels of these genes in the HI group. Based on all these findings mentioned above, it was indicated that 5 hmC modification may play a critical part in the pathogenesis of CP through regulating transcription and expression of the genes related to neuronal functions and Tuina may produce therapeutic effects on CP via modulating 5 hmC.

More interestingly, it was found that CP and related developmental disorders are more common seen in male patients [56, 57] and female neonatal mouse model of HI brain injury also displayed gender-specific lower occurrence of CP [58], the reason for which is still uncertain. In order to avoid the disruption from this possible gender-specific protective effects, only male rat pups were selected for our study. Remarkably, in genome-wide circular map (Figure 5(a)), great differences in the distribution of hyper-DhMRs and hypo-DhMRs on the X chromosome were observed from the comparison between any two groups in our study, which further confirms the correlation between sex and the occurrence of cerebral palsy. Notably, due to the limitations of our lab, we could not do IgG control or generate the input libraries, and DhMRs were identified by directly comparing one to the other rather than comparing to the input, which we will intend to improve in our future studies to make the research more precise.

Furthermore, GO analyses of hypo-DhMRs-associated genes in the HI group showed that these genes are highly enriched in multiple signaling pathways related to neurodevelopment and neuronal functions. In our experiment, growth retardation and neurodevelopmental disorders appeared in the HI group in the form of weight loss, weakness in limb strength, and several neuronal reflex disorders which were improved by Tuina treatment to some extent. Moreover, from the results of the Morris water maze test and step-down avoidance test, Tuina improved learning and memory functions of hypoxic-ischemic rats significantly. These results indicate the correlation between the alteration in 5-hydroxymethylcytosine loci and neuronal development and furtherly suggests that Tuina therapy may influence brain developments and cerebral cognitive functions via modulating DNA hydroxymethylation.

In the present study, we provided genome-wide 5 hmC maps of control, HI, and Tuina groups and revealed alteration in DNA hydroxymethylation status and 5 hmC dynamic change after Tuina treatment, which may shed light on the possible therapeutic mechanism of Tuina for CP.

## Data Availability

The data used to support the findings of this study are available from the corresponding author upon request.
