# Corrigendum to “Tuina Massage Improves Cognitive Functions of Hypoxic-Ischemic Neonatal Rats by Regulating Genome-Wide DNA Hydroxymethylation Levels”

**DOI:** 10.1155/2020/9461372

**Published:** 2020-05-09

**Authors:** Yunpeng Zhang, Chao Gao, Danmei Chen, Cuiting Wang, Long Chen, Yaodong Zhang, Bing Li

**Affiliations:** ^1^Research Center for Clinical Medicine, Jinshan Hospital, Fudan University, Shanghai, China; ^2^Institute of Neurology, Academy of Integrative Medicine, Fudan University, Shanghai, China; ^3^Department of Rehabilitation, Henan Children's Hospital, Zhengzhou University, Zhengzhou, China; ^4^Department of Neurology, Jinshan Hospital, Fudan University, Shanghai, China; ^5^Department of Pediatrics, Henan Children's Hospital, Zhengzhou University, Zhengzhou, China

In the article titled “Tuina Massage Improves Cognitive Functions of Hypoxic-Ischemic Neonatal Rats by Regulating Genome-Wide DNA Hydroxymethylation Levels” [1], there were errors in the presentation of Figure 1 that are corrected as follows.

In Figure 1(f), mean ± SD was used rather than mean ± S.E.M as described in the methods. In order to be consistent, Figure 1(f) is corrected to use mean ± S.E.M.

Figures 1(a)–1(c) were originally circle diagrams, and the lines inside the circle could not be seen clearly. A linear diagram gives a clear view of the distribution of hDMRs on each chromosome and facilitates comparison between the three groups.

The bar chart in Figures 1(d) and 1(e) showed only *p* values. These are replaced with a series of bubble diagrams, which can display the degree of functional enrichment from three indicators: the depth of the color corresponds to the *p* value in the original figure, the size of the point is gene count, and the Rich factor on the *x*-axis indicates the proportion of genes that falls into this GO term.

Moreover, Figures 1(d) and 1(e) originally showed the HI group's lower hydroxymethylation-associated DhMRs compared with the control 5(f) and tuina 5(e) groups. This has been changed to higher hydroxymethylation-associated DhMRs, which the authors believe is more meaningful for the subsequent MAPK signalling pathway studies. Accordingly, the result description “As shown in Figures 1(d) and 1(e), GO enrichment analysis indicated that these hypo-DhMRs…” should be replaced as follows.

As shown in Figures 1(d) and 1(e), GO enrichment analysis indicated that these hyper-DhMRs in the HI group were found highly enriched on the functional pathways related to neurodevelopment and neuronal functions, including positive regulation of MAPK cascade, regulation of signal transduction by protein phosphorylation, and the MAPK cascade itself.

## Figures and Tables

**Figure 1:**
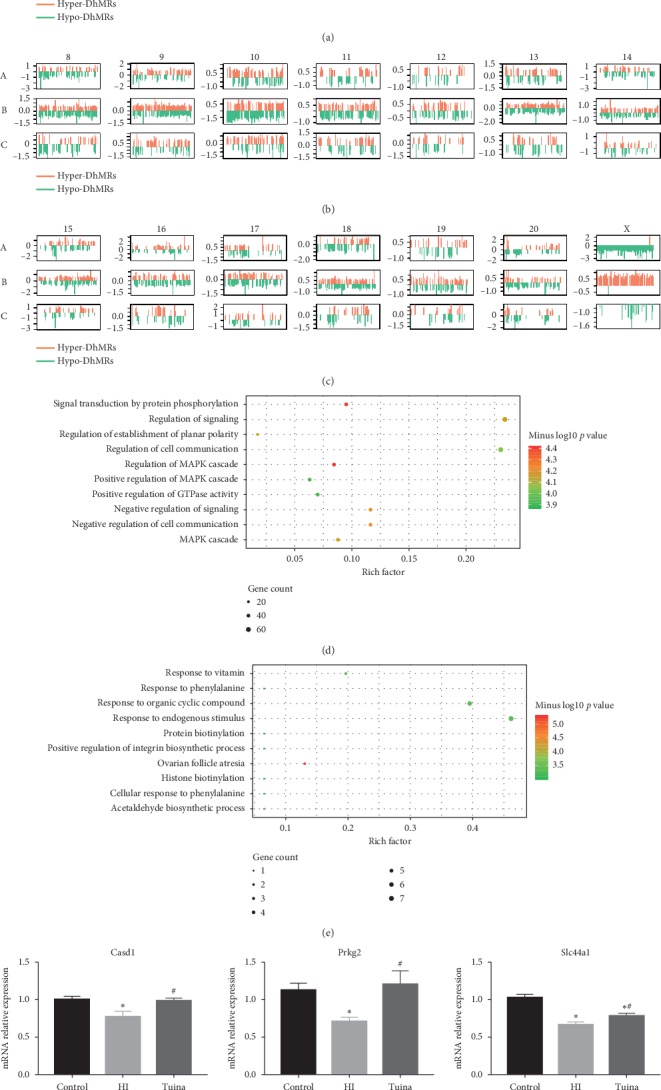
Identification and characterization of differentially hydroxymethylated regions (DhMRs). (a–c) Chromosome map of genome-wide DhMRs. (a) Control vs. HI group; (b) HI vs. tuina group; (c) control vs. tuina group. Top ten GO enrichment analysis results of HI group's higher hydroxymethylation associated DhMRs compared with the control (d) and tuina (e) group. (f) RT-PCR analysis of the mRNA levels of selected hypo-DhMRs related genes. *β*-Actin was used as a control. (^*∗*^*p* < 0.05 vs. control group and ^#^*p* < 0.05 vs. HI group).
